# Association of Reduction in Continuity of Care During COVID‐19 Pandemic With Cardiovascular Diseases, Kidney Failure and All‐Cause Mortality for People With Diabetes: A Cohort Study in Hong Kong

**DOI:** 10.1111/dom.70984

**Published:** 2026-06-15

**Authors:** Yuk Kam Yau, Meijiao Li, Jianchao Quan, Karen Ann Grépin, Gary Kui Kai Lau, Ivy Lynn Mak, Chak Sing Lau, Ian Chi Kei Wong, David Vai Kiong Chao, Welchie Wai Kit Ko, Cindy Lo Kuen Lam, Eric Yuk Fai Wan

**Affiliations:** ^1^ Department of Family Medicine and Primary Care, School of Clinical Medicine Li Ka Shing Faculty of Medicine, The University of Hong Kong Hong Kong China; ^2^ School of Public Health Li Ka Shing Faculty of Medicine, The University of Hong Kong Hong Kong China; ^3^ Department of Medicine, School of Clinical Medicine Li Ka Shing Faculty of Medicine, The University of Hong Kong Hong Kong China; ^4^ Department of Pharmacology and Pharmacy Li Ka Shing Faculty of Medicine, The University of Hong Kong Hong Kong China; ^5^ Aston Pharmacy School Aston University Birmingham UK; ^6^ Advanced Data Analytics for Medical Science Limited Hong Kong Special Administrative Region China; ^7^ Macau University of Science and Technology Macau Special Administrative Region China; ^8^ Department of Family Medicine and Primary Health Care United Christian Hospital, Kowloon East Cluster, Hospital Authority Hong Kong Special Administrative Region China; ^9^ Department of Family Medicine and Primary Healthcare Hong Kong West Cluster, Hospital Authority Hong Kong Special Administrative Region China; ^10^ Department of Family Medicine The University of Hong Kong‐Shenzhen Hospital Shenzhen China; ^11^ Comprehensive Primary Healthcare Collaboratory Li Ka Shing Faculty of Medicine, The University of Hong Kong Hong Kong Special Administrative Region China; ^12^ The Institute of Cardiovascular Science and Medicine, Li Ka Shing Faculty of Medicine, The University of Hong Kong Hong Kong Special Administrative Region China

**Keywords:** cardiovascular disease, continuity of care, death, kidney failure, type 2 diabetes

## Abstract

**Introduction:**

Continuity of care, a critical component of high‐quality primary care, was interrupted in people with diabetes mellitus during the COVID‐19 pandemic in Hong Kong. Research on whether reduced continuity of care was associated with higher complications and mortality risks is lacking.

**Methods:**

This retrospective cohort study included 123 403 eligible adult patients with diabetes. Continuity of care was assessed by the usual provider continuity index (UPCI), calculated as the proportion of attendances to the patients' most visited clinic during both pre‐outbreak period (August 2017–December 2019) and outbreak period (January 2020–May 2022). Patients were followed from 1 June 2022 until the outcomes of interest or June 2024. Cox regression was applied to evaluate the association between reduced continuity of care and risks of incident cardiovascular diseases (CVD), kidney failure and all‐cause mortality.

**Results:**

Greater reductions in UPCI were associated with higher risks of CVD; the hazard ratios (HR, 95% CI) for 10%–20% reduction were 1.05 (0.97, 1.14), HR = 1.33 (1.14, 1.56) for 20%–30% reduction, and HR = 1.30 (1.14, 1.48) for 30% reduction when compared to patients experiencing ≤ 10% reduction in UPCI. Similar patterns were found for CVD subtypes, including coronary heart disease and heart failure. Reductions of 20%–30% and > 30.1% in UPCI were associated with higher kidney failure risks.

**Conclusion:**

Greater impairment in continuity of care was associated with increased incidences of CVD in diabetic patients. Our results emphasize the need for health policies to have contingency plans for maintaining continuity of primary care for patients with DM in future public health crises.

## 1 Introduction

As one of the most prevalent non‐communicable diseases, diabetes mellitus (DM) is a critical challenge in public health. The prevalence of DM in adults aged 20–79 years is predicted to rise from 10.5% (536.6 million people) in 2021 to 12.2% (783.2 million) by 2045 worldwide [1]. Patients with DM typically have an elevated risk of cardiovascular diseases (CVD), typically 2.3 times higher risk than those without [2]. To prevent or postpone the onset of CVD, international clinical guidelines recommend that people with DM undergo at least one comprehensive medical assessment annually [3, 4, 5]. In Hong Kong, patients with DM are mainly managed in the primary care clinics [6].

Continuity of care is a critical hallmark of primary care, encompassing the three aspects of relational, informational, and management continuity in care provision over time [7]. Higher continuity of care achieves benefits for patients through improved trust between patients and healthcare providers, information transmission, disease monitoring, medication management, and adherence. Prior studies showed that better continuity of care was associated with lower incidence rates of complications and deaths in individuals with DM [8, 9, 10].

Continuity in primary care among diabetic patients was influenced worldwide throughout the coronavirus disease 2019 (COVID‐19) period [11, 12, 13]. In Hong Kong, some general outpatient clinics (GOPCs) were designated as COVID‐19 clinics to streamline resources and reduce the transmission of the virus during the COVID‐19 pandemic, but this may have negative consequences on the continuity of primary care for patients with DM [14]. There is a lack of evidence on whether a reduction in continuity of care during the pandemic increased the incidence of CVD, kidney failure, and mortality among patients with DM.

This study aimed to evaluate the effect of a reduction in continuity of care on the incidence of CVD, kidney failure, and mortality in diabetic patients. The findings can guide policymakers on the importance of implementing contingency plans for primary care provision in future public health emergencies.

## 2 Methods

### 2.1 Study Design and Population

**FIGURE 1:**
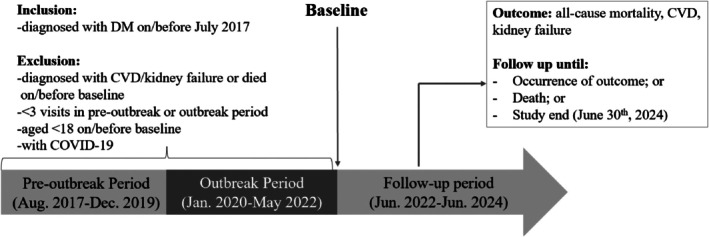
Study design. CVD, cardiovascular diseases; DM, diabetes mellitus; UPCI, usual provider continuity index.

This retrospective cohort study utilized electronic health records obtained from the Hospital Authority (HA), the sole provider of public healthcare services in Hong Kong. Data from the 2020–2022 Population Health Survey indicated that public clinics and hospitals in Hong Kong are the main care providers managing 86% of patients with DM [15]. Clinical information, including demographics, clinical parameters, diagnoses, medication use, and healthcare service utilization are recorded. The study included adults with DM from January 2010 to July 2017, identified using the International Classification of Primary Care (ICPC) and the International Classification of Diseases, Ninth Revision, Clinical Modification (ICD‐9‐CM) (Table S1). To validate that diagnosis codes are sufficient to capture diabetic patients, we conducted an analysis by expanding our identification criteria to include both prescription records of glucose‐lowering drugs and laboratory results (i.e., at least two HbA1c measurements of 6.5% or higher) [16]. Among the 128 779 patients identified through the expanded criteria, 123 665 (96.0%) were already captured by the ICD‐9‐CM and ICPC codes alone. Therefore, we believe we should have included the vast majority of patients with DM managed in the Hong Kong public healthcare system. Exclusion criteria include: (1) having a diagnosis of CVD or kidney failure on or before May 2022; (2) having fewer than three clinic visits during the pre‐outbreak or outbreak periods to minimize the biases by the high UPCI scores among patients with small number of visits; (3) having an increased usual provider continuity index (UPCI) change; (4) having a confirmed diagnosis of COVID‐19 or drug records of the treatment for COVID‐19 from January 2020 to June 2024 to exclude the direct biological impact of the SARS‐CoV‐2 virus. The baseline was set at 1 June 2022. Individuals were followed from 1 June 2022 until the date of death, the outcome of interest, or the study end (June 2024), whichever came first. Figure 1 shows the detailed study design.

### 2.2 Measurement of the Changes in Continuity of Care

Continuity of care was measured using clinic‐level UPCI, calculated as the number of attendances to the patients' most frequently visited clinic divided by their total number of attendances. The exact formula for UPCI is: $\text{UPCI}=\frac{\text{Number of attendances to the most frequently visited clinic}}{\text{Total number of visits}}\times 100\%$. The UPCI is a widely used and valid measure of continuity of care [17, 18]. The UPCI score ranges from 0 to 1. A score of 0.5 indicates patients visited the same clinic for half of all attendances. The percentage change in UPCI was calculated as the difference between the UPCI during the outbreak period and the pre‐outbreak period, divided by the UPCI during the pre‐outbreak period. Attendance was counted for visits to general outpatient clinics (GOPCs) provided publicly and under the public‐private partnership programs. Visits to a private physician under the public‐private partnership programs are subsidized by the government [19]. In Hong Kong, telehealth services were introduced during the COVID‐19 pandemic period, and teleconsultations provided by primary care clinics were also counted as attendances in our analyses. In Hong Kong, the outbreak period began in January 2020 (first confirmed COVID‐19 case) and ended in May 2022 when the Omicron outbreak subsided. The pre‐outbreak period spanned an equivalent duration as the outbreak period from August 2017 to December 2019.

### 2.3 Outcomes

The primary outcome of interest was incident CVD, that is, any events of coronary heart disease (CHD), heart failure (HF), or stroke. Secondary outcomes included all‐cause mortality and kidney failure. CVD events were defined based on ICD‐9‐CM codes or ICPC‐2 codes. Kidney failure was identified using ICD‐9‐CM codes or an estimated glomerular filtration rate (eGFR) below 15 mL/min/1.73 m^2^. The detailed case definition is shown in Table S1.

### 2.4 Baseline Characteristics

We constructed a directed acyclic graph (DAG) using the web application DAGitty v3.1 (https://dagitty.net/) to illustrate the assumed pathways between the usual provider continuity index (UPCI) reduction (the exposure) and CVD (the primary outcome) among patients with DM (Figure S1). Based on previous studies and DAG, the minimally sufficient adjustment set was determined. Baseline characteristics were defined as the most recent measurement within 2 years before baseline. Baseline covariates included sex, age, smoking (yes or no), Charlson Comorbidity Index (CCI), body mass index (BMI), systolic blood pressure (SBP), diastolic blood pressure (DBP), glycated haemoglobin (HbA1c), low‐density lipoprotein cholesterol and estimated glomerular filtration rate (eGFR), use of antihypertensive drugs, insulin, oral antidiabetic drugs, and lipid‐lowering drugs, UPCI during the pre‐outbreak period and the number of healthcare service attendances, including specialist outpatient clinics (SOPC), accident & emergency (A&E), and hospital admission. The baseline characteristics were systematically captured for all patients.

### 2.5 Statistical Analysis

Patients with DM were categorized into four groups based on the percentage of reduction in their UPCI: (1) ≤ 10.0% (reference group); (2) 10.1%–20.0%; (3) 20.1%–30.0%; and (4) ≥ 30.1%. The UPCI reduction categories were defined based on clinical relevance. In our cohort of patients with DM, the average number of primary care visits during the pre‐outbreak period was approximately 10. Therefore, a 10% reduction in UPCI corresponds approximately to a change of one visit from a patient's usual provider. A reduction below 10% represents a normal year‐to‐year fluctuation in visit patterns rather than a true deterioration in continuity. We used equal‐width intervals (10% increments) for other cut‐points. By using 10% increments, we provide a clinically intuitive gradient: each successive group represents a further decrease in continuity of approximately one encounter with a familiar clinic, which is easier for policymakers and clinicians to understand and interpret. To reduce potential confounding and enhance group comparability, fine stratification weights were employed [20]. This approach created strata based on the propensity score prior to calculating weights within each stratum. The propensity scores were estimated using multivariable logistic regression incorporating baseline characteristics mentioned above. The balance of baseline covariates across groups was assessed using standardized mean differences (SMD), with an SMD < 0.2 indicating sufficient balance across groups after weighting. Missing baseline data were handled through multiple imputation, with each missing variable imputed five times using chained equations based on other known baseline variables; results were combined using Rubin's rules. Multivariable Cox proportional hazards models, adjusted for baseline covariates, were used to estimate hazard ratios (HR) and 95% confidence intervals (CI). We conducted subgroup analyses stratified by the sex, age (< 65; ≥ 65 years), CCI (< 4, ≥ 4), smoking (Yes; No), HbA1c (< 7, ≥ 7%), blood pressure (SBP < 130 and DBP < 80 mmHg, SBP ≥ 130 or DBP ≥ 80 mmHg), BMI (< 25, ≥ 25 kg/m^2^), eGFR (< 60, ≥ 60 mL/min/1.73 m^2^). Several sensitivity analyses on outcomes of interest were performed: (1) performing a competing risk analysis with the Fine‐Gray model, to account for potential death as a competing risks of death event; (2) including only patients with at least five attendances during both the pre‐outbreak and outbreak periods; (3) using inverse probability of treatment weighting (IPTW), rather than fine stratification weights; (4) using UPCI changes (per 10% decrease) as a continuous variable; (5) defining kidney failure using at least two eGFR measurements < 15 mL/min/1.73 m^2^ recorded and ICD‐9‐CM codes; (6) adjusting HbA1c, blood pressure, and medication use as time‐varying confounders using a time‐dependent Cox proportional hazards model; (7) including all patients, regardless of COVID‐19 status, while adjusting for COVID‐19 infection as a covariate to address the potential collider bias introduced by excluding patients with confirmed COVID‐19 infection; (8) using pre‐outbreak variables to construct propensity scores to address potential violation of temporality; (9) removing healthcare utilization during the outbreak period in the adjustment sets to address potential over‐adjustment and collider bias; (10) excluding individuals with diagnosis codes of pneumonia, cough, and upper respiratory infection, as patients with pneumonia, cough and upper respiratory infection diagnosis codes could be COVID‐19 cases during the pandemic [21, 22].

All statistical analyses were performed in Stata 15.1 software and R version 4.5.1. All tests were two‐tailed, and a *p*‐value of less than 0.05 was considered statistically significant.

## 3 Results

### 3.1 Patient Characteristics

A total of 123 665 patents were identified. Baseline characteristics of patients before weighting are shown in Table S2. Patients who had larger reductions in UPCI tended to be younger, had higher baseline HbA1c levels, were more likely to use insulin, had lower UPCI during the pre‐outbreak period and attended A&E and SOPC more frequently. After weighting, 262 participants were excluded due to the absence of suitable matches (Figure S2). Consequently, a total of 123 403 eligible patients were included in the analysis, with 67 391 (54.6%) being female and a mean age of 69.0 years (Table 1). The proportion of patients by UPCI changes, with each increment of 10%, is shown in Table S3. The completion rates of the baseline characteristics range from 88.1% to 98.6%, with an overall completion rate of 86.6% (Table S4). All standardized mean differences (SMDs) were < 0.2 after weighting, suggesting that the groups were well‐balanced.

**TABLE 1:** Baseline characteristics of patients with DM stratified by clinic‐based UPCI reduction.

Characteristics	Total (*N* = 123 403)	UPCI reduction, (≤ 10%, *N* = 102 972) (Reference)	UPCI reduction, (10.1%–20%, *N* = 13 549)	UPCI reduction, (20.1%–30%, *N* = 2870)	UPCI reduction, (≥ 30.1%, *N* = 4012)	SMD
Age, years	69.00 ± 10.25	68.95 ± 10.21	69.06 ± 10.33	69.38 ± 10.45	69.63 ± 10.76	0.07
Sex, female	67 391 (54.61)	56 147 (54.53)	7405 (54.66)	1581 (55.09)	2258 (56.27)	0.04
CCI	3.78 ± 1.42	3.78 ± 1.41	3.78 ± 1.43	3.81 ± 1.42	3.85 ± 1.46	0.05
Smoking	36 897 (29.90)	30 872 (29.98)	4064 (29.99)	822 (28.65)	1140 (28.42)	0.03
Clinical parameters						
HbA1c (%)	6.97 ± 0.96	6.97 ± 0.96	6.96 ± 0.92	6.94 ± 0.93	6.92 ± 0.93	0.05
SBP (mmHg)	132.04 ± 14.45	132.04 ± 14.44	131.99 ± 14.23	131.95 ± 14.63	132.14 ± 15.23	0.01
DBP (mmHg)	70.59 ± 10.34	70.63 ± 10.34	70.46 ± 10.38	70.45 ± 10.12	70.27 ± 10.38	0.03
eGFR (mL/min/1.73 m^2^)	78.35 ± 18.79	78.40 ± 18.77	78.22 ± 18.82	77.67 ± 18.85	77.83 ± 19.11	0.04
BMI (kg/m^2^)	25.57 ± 4.25	25.57 ± 4.24	25.56 ± 4.21	25.71 ± 4.42	25.50 ± 4.32	0.05
LDL‐C (mmol/L)	2.06 ± 0.59	2.06 ± 0.59	2.06 ± 0.59	2.04 ± 0.60	2.05 ± 0.62	0.04
Medicine use						
Lipid lowering drugs	100 366 (81.33)	83 736 (81.32)	11 029 (81.40)	2333 (81.29)	3269 (81.47)	0.00
Antihypertensive drugs	104 880 (84.99)	87 438 (84.91)	11 546 (85.21)	2475 (86.23)	3421 (85.27)	0.04
Insulin	10 355 (8.39)	8724 (8.47)	1115 (8.23)	221 (7.69)	296 (7.38)	0.04
Oral antidiabetic drugs	111 778 (90.58)	93 273 (90.58)	12 251 (90.42)	2614 (91.08)	3640 (90.73)	0.02
UPCI, August 2017–December 2019	0.99 ± 0.05	0.99 ± 0.05	0.99 ± 0.05	0.99 ± 0.05	0.99 ± 0.05	0.05
Number of hospital admissions, January 2020–May 2022	0.56 ± 1.87	0.56 ± 1.89	0.54 ± 1.80	0.53 ± 1.51	0.58 ± 1.64	0.04
Number of A&E attendance, January 2020–May 2022	0.55 ± 1.34	0.55 ± 1.38	0.54 ± 1.13	0.56 ± 1.14	0.64 ± 1.23	0.09
Number of SOPC attendance, January 2020–May 2022	0.70 ± 2.00	0.70 ± 2.02	0.68 ± 1.98	0.65 ± 1.81	0.69 ± 1.90	0.02

*Note:* Data are generated after multiple imputation and weighting. Data are expressed in mean ± SD or number (percentage) as appropriate.

Abbreviations: A&E, accident & emergency; BMI, body mass index; CCI, Charlson Comorbidity Index; DBP, diastolic blood pressure; DM, diabetes mellitus; eGFR, estimated glomerular filtration rate; GOPC, general outpatient clinic; HbA1c, glycated haemoglobin; LDL‐C, low density lipoprotein—cholesterol; SBP, systolic blood pressure; SMD, standardized mean difference; SOPC, specialist outpatient clinic; UPCI, usual provider continuity index.

### 3.2 Associations of UPCI Reductions With All‐Cause Mortality, CVD and Kidney Failure

**FIGURE 2:**
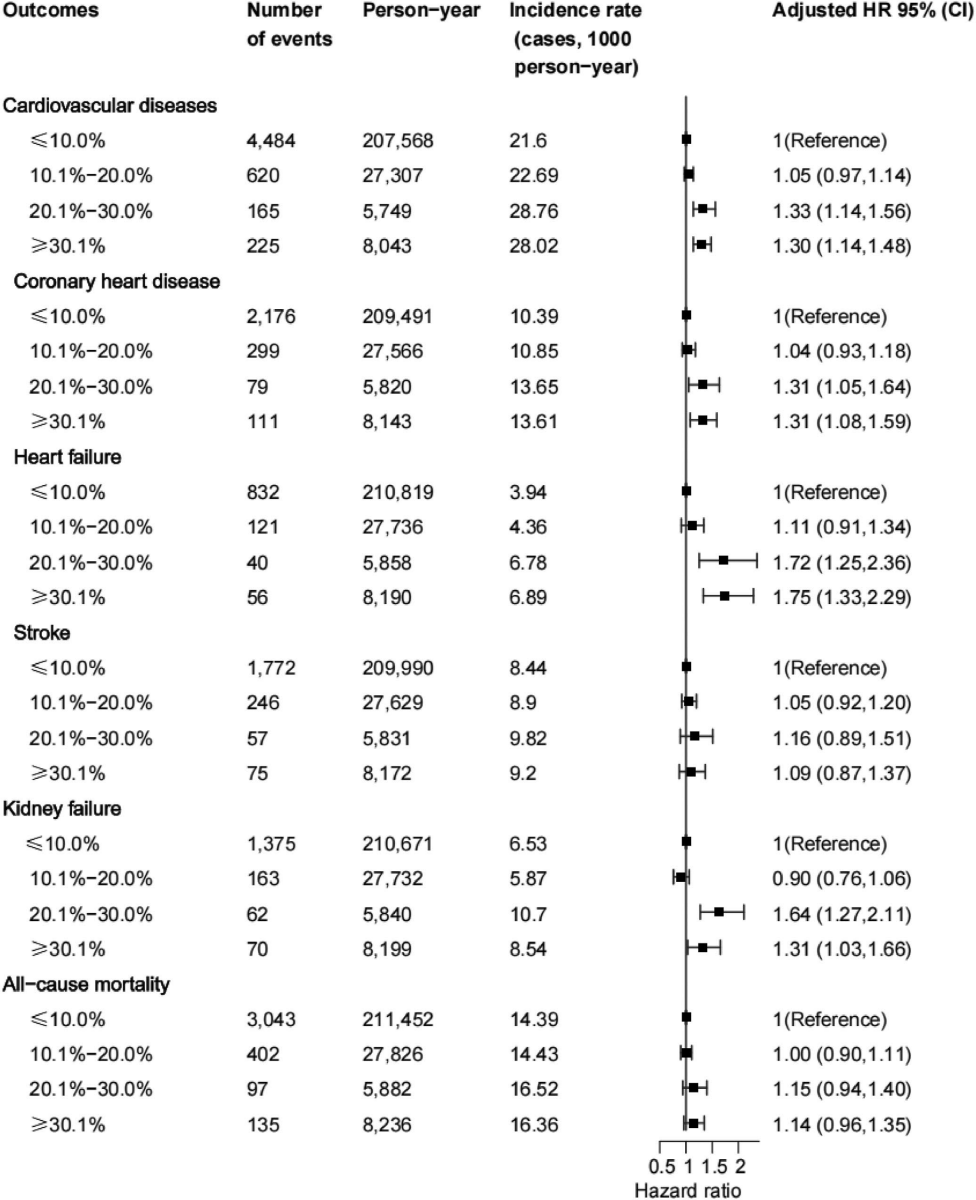
Associations of clinic‐based UPCI reduction with CVD, kidney failure and all‐cause mortality among patients with DM. The analysis was conducted after applying multiple imputation and weighting to the data. Hazard ratios were adjusted for age, sex, Charlson Comorbidity Index, smoking, systolic blood pressure, diastolic blood pressure, glycated haemoglobin, estimated glomerular filtration rate, body mass index, low‐density lipoprotein cholesterol, insulin, oral antidiabetic drugs, lipid lowering drugs, antihypertensive drugs, UPCI (August 2017–December 2019), number of hospital admissions (January 2020–May 2022), number of A&E attendances (January 2020–May 2022) and number of SOPC attendances (January 2020–May 2022). CVD, cardiovascular diseases; DM, diabetes mellitus; GOPC, general outpatient clinic; UPCI, usual provider continuity index.

Over a median follow‐up period of 2.1 years (248 667 person‐years), the HR (95% CI) for overall CVD of patients with UPCI reduction by 10.1%–20.0%, 20.1%–30%, ≥ 30.1%, relative to ≤ 10% were 1.05 (0.97, 1.14), 1.33 (1.14, 1.56), and 1.30 (1.14, 1.48), respectively (Figure 2). CHD and HF exhibited consistent patterns, but stroke did not. Patients with UPCI reductions by 20.1%–30.0% and ≥ 30.1% had higher risks of kidney failure compared to those with UPCI reductions by ≤ 10.0%, with HR (95% CI) of 1.64 (1.27, 2.11) and 1.31 (1.03, 1.66), respectively. There was no significant difference in the risks of all‐cause mortality across the groups.

### 3.3 Subgroup and Sensitivity Analysis

Subgroup analyses of the CVD outcome are presented in Figure S3. The associations between UPCI reductions and increased risks of CVD were stronger in individuals with higher eGFR (eGFR ≥ 60) and SBP ≥ 130 mmHg or DBP ≥ 80 mmHg at baseline than those with lower eGFR (eGFR < 60) and SBP < 130 mmHg and DBP < 80 mmHg, respectively. Results from the sensitivity analyses showed similar associations between UPCI reductions and the risks of CVD (Tables S5–S14).

## 4 Discussion

The findings of this large‐scale cohort study suggested that a larger reduction in continuity of care was associated with increased risks of CVD and kidney failure in individuals with DM. Similar associations were observed for CHD and HF, but were less apparent for stroke and all‐cause mortality. The results suggest that contingency plans to ensure the continuity of care are needed, such as teleconsultations, among patients with DM in future public health crises.

Previous studies have shown that better continuity of care was associated with reduced risks of CVD and kidney failure in patients with DM. In a prior cohort study in Hong Kong, compared with patients in the lowest quartile of continuity of care, those in the 2nd to 4th quartiles were 5% to 13% less likely to develop overall CVD [8]. Another cohort study in patients with newly diagnosed type 2 DM reported that the intermediate and low continuity of care groups had greater risks of incident kidney failure than the high continuity group [23]. Studies showed that the COVID‐19 pandemic has influenced both number of visits to primary care and continuity of care for patients with DM [13, 24], but these studies did not examine the association of reduced continuity of care with the CVD risks. This study contributes additional evidence to the existing body of literature on the relative risk of reduction in continuity of care associated with CVD and kidney failure in diabetic patients.

Since the COVID‐19 pandemic started, some primary care clinics were designated as COVID‐19 clinics in Hong Kong, which affected the continuity of primary care for DM. Evidence has shown that better continuity of care is associated with higher patient satisfaction [25, 26] and improved patient‐physician relationship [27], thereby facilitating better communication between patients and their physicians and enhancing medication adherence. In addition, through high continuity of care, physicians can accumulate information about the patient, arrange necessary examinations and laboratory tests, and provide appropriate medications [28, 29]. Studies have reported that higher continuity of care was associated with better glycemic, blood pressure, and lipid control, which are the risk factors for CVD [30, 31, 32, 33]. The reduced continuity of care could undermine the patient‐physician relationship, potentially reducing patients' adherence to lifestyle advice and treatment as well as affecting the physician's decisions on medication and examination arrangements, thereby increasing the risks of CVD. The lack of a significant association between reduced continuity of care and stroke may be explained by the diverse etiologies of stroke in patients with DM. Patients with diabetes are susceptible to cerebral small vessel diseases (CSVD), one of the main reasons contributing to stroke. However, while modifiable risk factor management is one of the main pathways through which high continuity of care benefits patients, studies suggest that CSVD may be less sensitive to traditional metabolic management, such as LDL‐C control, making the effect of reduced continuity of care weaker for stroke [34, 35]. Therefore, the reduced continuity of care during the pandemic has a weaker effect on stroke.

In this study, we did not observe a significant association between reduced continuity of care and increased risks of all‐cause mortality. This may be attributable to the limited follow‐up duration, which was likely insufficient for the effect of reduced continuity of care on all‐cause mortality to manifest. Future studies with a longer follow‐up period are needed to evaluate the association of the reduction in continuity of care during the pandemic with all‐cause mortality in patients with DM. Another possible explanation for the absence of a significant association could be attributed to the dilution effect of deaths not related to CVD. The top three causes of death in our cohort were malignant neoplasms (29.2%), lower respiratory infections (26.0%) and CVDs (17.3%). While reduced continuity of care in primary care directly impacts the management of DM, it has a less immediate or direct influence on mortality from cancer or respiratory infections. Given that over 55% of causes of death in our cohort were not related to CVD, the specific protective effect of continuity of care on cardiovascular health was not sufficient to shift the trend for all‐cause mortality within the study period.

We observed stronger associations between the reduction in continuity of care and increased CVD incidence rates in subgroups with suboptimal SBP or DBP control and those with better kidney function (eGFR > 60) at baseline. Previous studies have demonstrated that high continuity of care helps patients achieve the blood pressure control target [30]. When continuity of care was reduced, patients with unsatisfactory blood pressure control may have a lower likelihood of achieving the blood pressure target, consequently increasing their CVD incidence rates. Individuals with impaired kidney function were more likely to experience worsening health status and have elevated CVD risks. Continuing follow‐up with the usual provider may contribute to a limited effect on reducing CVD incidence rates for these patients.

While relational continuity focuses on the continuous relationship between a patient and one or more providers, clinic‐level continuity measured in this study reflects the stability of the care environment. In the Hong Kong public healthcare system, care for patients with DM is provided by a multi‐disciplinary team within a general outpatient clinic (GOPC). Maintaining clinic‐level continuity of care enables patients to be managed by a familiar clinical team with integrated health records and shared management protocols. Our findings suggest that during the pandemic, the maintenance of clinic‐based relational continuity of care is important in the management of DM. In healthcare systems where individual provider‐level continuity is difficult to implement (e.g., due to doctor rotation, high patient volumes), clinic‐level continuity may be a feasible policy target. Future studies are needed to measure both clinic‐level and provider‐level continuity to determine their independent contributions.

To our knowledge, this is the first study to examine the association of the reduction of continuity of care during the COVID‐19 pandemic with the incidence of CVD, kidney failure and mortality among patients with DM. This study has several strengths, including the analysis of a large‐scale, population‐based cohort of over 120 000 people with DM in Hong Kong before and during the pandemic. In addition, this study evaluated the associations in subgroup analyses, providing a nuanced understanding of the effects of the reduction in continuity of care on this patient population with various baseline characteristics. The findings of this study provide insights into the clinical management of people with DM during future public health crises. Our results suggested that contingency plans to maintain continuity of primary care are needed for patients with DM, to ensure the quality of care for this vulnerable population when reorganizing the healthcare resources during acute public health crises. Nevertheless, there were some limitations in this study. First, this study has a relatively short follow‐up period and may not capture the long‐term effects of the reduction in continuity of care. Second, since the analysis relied on data derived from the healthcare system in Hong Kong, the findings may not be generalizable to other countries or districts with different healthcare settings. Third, due to the unavailability of the data specifying the physician seen at each primary care attendance, the UPCI was calculated at clinic level and the relational continuity may not be fully captured. Fourth, data from rapid antigen tests (RAT) were unavailable in this study, which may potentially contribute to incomplete COVID‐19 case capture. However, undetected or RAT test‐positive cases without PCR confirmation were likely to be mild infections that did not require hospitalization. COVID‐19 cases that would affect health service use were likely to be more serious cases, who would have been diagnosed by PCR and excluded from this study. Furthermore, all patients with major CVD events and kidney failure would have been admitted to the hospital, where PCR for COVID‐19 was a routine. Thus, the possible bias from missed mild COVID‐19 cases on the CVD rates was minimal. Additionally, we conducted a sensitivity analysis excluding individuals with diagnosis codes of pneumonia, cough, and upper respiratory infection, which could be COVID‐19 cases during the pandemic. The results for overall CVD, CVD subtypes and kidney failure remained consistent with the main analysis. Fifth, the measurement of UPCI change required patients to survive throughout the outbreak period, which potentially introduces survivor bias. However, we observed that after applying other exclusion criteria (i.e., those with COVID‐19, pre‐existing CVD or kidney failure prior to baseline, or less than 3 visits in pre‐outbreak or outbreak period), only 2541 out of 537 405 (0.47%) patients were excluded due to death on or before May 2022. Given that these patients represent a small fraction of the overall cohort, the potential for survivor bias and immortal time bias should be minimal in this study. Finally, the measure of reduction of continuity of care by UPCI had limitations in that it could not differentiate patient‐level healthcare‐seeking behaviour changes from system‐level healthcare reallocations. Future studies are needed to disentangle these specific causal pathways.

## 5 Conclusion

A higher reduction in continuity of care during the COVID‐19 pandemic was associated with elevated incidences of overall CVD among diabetic patients. Our findings inform healthcare policy that contingency plans to maintain the continuity of primary care are needed among patients with DM in future public health crises.

## Author Contributions

Study design and conception: Y.K.Y., E.Y.F.W. Data acquisition: Y.K.Y., E.Y.F.W. Statistical analysis: M.L. Data interpretation: Y.K.Y., C.L.K.L., E.Y.F.W. Manuscript draft: Y.K.Y. Critical revision: Y.K.Y., J.Q., K.A.G., G.K.K.L., I.L.M., C.S.L., I.C.K.W., D.V.K.C., W.W.K.K., C.L.K.L., E.Y.F.W. Funding acquisition: C.L.K.L., E.Y.F.W. Supervision or mentorship: C.L.K.L., E.Y.F.W. All authors read and approved the manuscript for submission.

## Funding

This work was funded by the Health and Medical Research Fund, Health Bureau, Government of Hong Kong Special Administrative Region (grant no: COVID19F08).

## Disclosure

The funder had no role in the design and conduct of the study; collection, management, analysis, and interpretation of the data; and preparation of the manuscript. During the preparation of this work, the author(s) did not use any generative AI tools.

## Ethics Statement

This study was approved by the Severance Hospital Institutional Review Board (IRB No. 4‐2022‐0248) and the Institutional Review Board of the University of Hong Kong Hospital Authority Hong Kong West Cluster (ref.: UW 21‐297).

## Consent

All extracted data is anonymous and no patient personal contact will be required and thus posing minimal ethical issues. Consent to participate was not required as the data used in this study are anonymized.

## Conflicts of Interest

E.Y.F.W. has received research grants from the Health Bureau, the Hong Kong Research Grants Council, Narcotics Division, Security Bureau, Social Welfare Department, Labour and Welfare Bureau of the Government of the Hong Kong SAR and National Natural Science Foundation of China; serves as a member of Core Team for Expert Group on Drug Registration of Pharmacy and Poisons Board, and is the director of Advance Data Analytics for Medical Science (ADAMS) Limited (HK). These are outside the submitted work. I.C.K.W. received research grants outside of submitted work from European Commission, Research Grants Council of the Hong Kong Special Administrative Region of the People’s Republic of China, Health and Medical Research Fund of the Government of the Hong Kong Special Administrative Region of the People’s Republic of China and Amgen; consulting fees from IQVIA and World Health Organization; served as a member of Pharmacy and Poisons Board of Hong Kong; was the founder and director of Therakind Limited (UK). He is the non‐executive director of Jacobson Pharma Corp. Ltd. in Hong Kong; and director of Advance Data Analytics for Medical Science (ADAMS) Limited (HK), Healthcare Innovation Technology Service (HITS) limited (UK) and OCUS Innovation Limited (HK, Ireland and UK). He received honorarium from Takeda as a speaker of continuous professional education study day. C.L.K.L. has received research grants from the Health Bureau of the Government of the Hong Kong SAR, conference grants from the Hong Kong College of Family Physicians and honoraria from the World Organization of Family Doctors, the Academy of Family Physicians of Malaysia and the International Association of Chinese Nephrologists, outside the submitted work.

## Supporting information


**Table S1:** Definition of the diseases.
**Table S2:** Baseline characteristics of patients with DM stratified by clinic‐based UPCI reduction after multiple imputation without weighting.
**Table S3:** The Proportion of Patients by UPCI Changes (*N* = 123 665).
**Table S4:** Data Completion Rates of the Baseline Characteristics among Patients with DM (*N* = 123 665).
**Table S5:** Associations between Clinic‐Based UPCI Reduction and CVD, Kidney Failure and All‐Cause Mortality among Patients with DM in Sensitivity Analysis of Using Inverse Probability of Treatment Weighting (*N* = 124 002).
**Table S6:** Associations of Clinic‐Based UPCI Reduction with CVD and Kidney Failure among Patients with DM in Sensitivity Analysis using Competing Risk Model (*N* = 123 403).
**Table S7:** Associations of Clinic‐Based UPCI Reduction with CVD, Kidney Failure and All‐Cause Mortality among Patients with DM in Sensitivity Analysis of including patients with at least five GOPC Attendance (*N* = 115 844).
**Table S8:** Associations of Clinic‐Based UPCI Reduction with CVD, Kidney Failure and All‐Cause Mortality among Patients with DM in Sensitivity Analysis of using UPCI Changes (per 10% decrease) as a Continuous Variable (*N* = 120 008).
**Table S9:** Associations of Clinic‐Based UPCI Reduction with Kidney Failure among Patients with DM in Sensitivity Analysis of Using at Least Two eGFR Measurements < 15 mL/min/1.73 m^2^ (*N* = 123 403).
**Table S10:** Associations of Clinic‐Based UPCI Reduction with CVD, Kidney Failure and All‐Cause Mortality among Patients with DM in Sensitivity Analysis of adjusting HbA1c, Blood Pressure, and Medication Use as Time‐varying Confounders (*N* = 123 403).
**Table S11:** Associations of Clinic‐Based UPCI Reduction with CVD, Kidney Failure and All‐Cause Mortality among Patients with DM in Sensitivity Analysis of Including Patients with COVID‐19 (*N* = 160 732).
**Table S12:** Associations of Clinic‐Based UPCI Reduction with CVD, Kidney Failure and All‐Cause Mortality among Patients with DM in Sensitivity Analysis of Using Pre‐outbreak Variables to Construct Propensity Scores (*N* = 123 429).
**Table S13:** Associations of Clinic‐Based UPCI Reduction with CVD, Kidney Failure and All‐Cause Mortality among Patients with DM in Sensitivity Analysis of Removing Healthcare Utilization during the Outbreak Period in the Adjustment Sets (*N* = 123 416).
**Table S14:** Associations of Clinic‐Based UPCI Reduction with CVD, Kidney Failure and All‐Cause Mortality among Patients with DM in Sensitivity Analysis of Excluding Individuals Diagnosed with Pneumonia, Cough, and Upper Respiratory Infection (*N* = 121 243).

**Figure S1:** Directed acyclic graph based on the assumed pathways.
**Figure S2:** Flowchart of patient selection.
**Figure S3:** Subgroup analyses on the association between the UPCI reduction and cardiovascular diseases among patients with DM.

## Data Availability

Data will not be made available to others because the data custodians have not given permission.
