# Supplementary material for: Association of Reduction in Continuity of Care During COVID‐19 Pandemic With Cardiovascular Diseases, Kidney Failure and All‐Cause Mortality for People With Diabetes: A Cohort Study in Hong Kong
Source: Diabetes Obes Metab. 2026 Jun 15;28(9):8016–24. doi: 10.1111/dom.70984 (PMC13449042; doi:10.1111/dom.70984)
Supplement: Supplementary file 1 [file DOM-28-8016-s001.docx]

**Table S1 Definition of the diseases**

| **Disease** | | **ICPC-2** | **ICD-9-CM** | **Others** |
| --- | --- | --- | --- | --- |
| **DM** | | T89-T90 | 250 | NA |
| **Kidney failure** | | NA | 585.5, 585.6, 586, V42.0, V56, 39.95, 54.98, 55.6 | eGFR <15 mL/min/1.73 m^2^ |
| **CVD** | **CHD** | K74-K76 | 410-414, 429.79 | NA |
|  | **Stroke** | K89-K91 | 430-438 | NA |
|  | **HF** | K77 | 428, 402.01, 402.11, 402.91 | NA |
| **COVID-19** | | A77 | NA | Positive results from PCR testing, or administration of antiviral treatments of Paxlovid or Molnupiravir |

Notes: ICPC-2, the International Classification of Primary Care-2; ICD-9-CM, the International Classification of Diseases, Ninth Edition, Clinical Modification; DM: diabetes mellitus; CVD, cardiovascular disease; CHD: coronary heart disease; HF: heart failure; eGFR, estimated glomerular filtration rate; NA, not applicable; PCR: polymerase chain reaction.

**Table S2 Baseline Characteristics of Patients with DM Stratified by Clinic-Based UPCI Reduction After Multiple Imputation Without Weighting**

| **Characteristics** | **Total  (N=123,665)** | **UPCI reduction, (≤ 10%, N=103,180)** | **UPCI reduction, (10.1%-20%, N=13,588)** | **UPCI reduction, (20.1%-30%, N=2,880)** | **UPCI reduction,  (≥30.1%, N=4,017)** | **SMD** |
| --- | --- | --- | --- | --- | --- | --- |
| Age, years | 69.01 ± 10.27 | 69.21 ± 10.17 | 68.56 ± 10.43 | 67.27 ± 10.79 | 66.74 ± 11.20 | 0.24 |
| Sex, female | 67,589 (54.65) | 56,773 (55.02) | 7,389 (54.38) | 1,465 (50.87) | 1,962 (48.84) | 0.12 |
| CCI | 3.78 ± 1.41 | 3.80 ± 1.40 | 3.75 ± 1.47 | 3.67 ± 1.52 | 3.67 ± 1.59 | 0.09 |
| Smoking | 36,961 (29.89) | 30,542 (29.60) | 4,033 (29.68) | 969 (33.65) | 1,417 (35.28) | 0.12 |
| Clinical parameters |  |  |  |  |  |  |
| HbA1c (%) | 6.97 ± 0.95 | 6.95 ± 0.93 | 7.00 ± 0.96 | 7.13 ± 1.18 | 7.20 ± 1.26 | 0.27 |
| SBP (mmHg) | 132.03 ± 14.44 | 132.02 ± 14.36 | 132.11 ± 14.44 | 132.17 ± 15.34 | 131.88 ± 15.71 | 0.02 |
| DBP (mmHg) | 70.56 ± 10.36 | 70.39 ± 10.31 | 71.25 ± 10.47 | 71.35 ± 10.55 | 72.04 ± 10.79 | 0.16 |
| eGFR (mL/min/1.73 m^2^) | 78.30 ± 18.83 | 78.04 ± 18.79 | 79.22 ± 18.72 | 80.41 ± 19.13 | 80.26 ± 19.75 | 0.13 |
| BMI (kg/m^2^) | 25.57 ± 4.24 | 25.56 ± 4.22 | 25.61 ± 4.26 | 25.59 ± 4.36 | 25.74 ± 4.50 | 0.04 |
| LDL-C (mmol/L) | 2.06 ± 0.59 | 2.05 ± 0.58 | 2.04 ± 0.59 | 2.14 ± 0.66 | 2.14 ± 0.67 | 0.16 |
| Medicine use |  |  |  |  |  |  |
| Lipid lowering drugs | 100,633 (81.38) | 84,174 (81.58) | 10,990 (80.88) | 2,277 (79.06) | 3,192 (79.46) | 0.06 |
| Antihypertensive drugs | 105,192 (85.06) | 88,187 (85.47) | 11,365 (83.64) | 2,356 (81.81) | 3,284 (81.75) | 0.10 |
| Insulin | 10,266 (8.30) | 8,054 (7.81) | 1,160 (8.54) | 398 (13.82) | 654 (16.28) | 0.26 |
| Oral antidiabetic drugs | 111,985 (90.56) | 93,418 (90.54) | 12,341 (90.82) | 2,609 (90.59) | 3,617 (90.04) | 0.03 |
| UPCI, August 2017-December 2019 | 0.99 ± 0.05 | 1.00 ± 0.03 | 0.98 ± 0.07 | 0.94 ± 0.11 | 0.95 ± 0.10 | 1.40 |
| Number of hospital admissions, January 2020-May 2022 | 0.55 ± 1.83 | 0.52 ± 1.76 | 0.60 ± 2.04 | 0.81 ± 2.27 | 0.85 ± 2.29 | 0.19 |
| Number of A&E attendance, January 2020-May 2022 | 0.54 ± 1.62 | 0.50 ± 1.25 | 0.63 ± 1.29 | 0.95 ± 2.17 | 1.10 ± 5.54 | 0.37 |
| Number of SOPC attendance, January 2020-May 2022 | 0.69 ± 1.97 | 0.66 ± 1.90 | 0.74 ± 2.09 | 1.03 ± 2.36 | 1.23 ± 2.71 | 0.30 |

Data are expressed in mean ± SD or number (percentage) as appropriate.

DM: diabetes mellitus; UPCI: Usual provider continuity index; SMD: Standardized mean difference; CCI: Charlson Comorbidity Index; HbA1c: Glycated hemoglobin; SBP: Systolic blood pressure; DBP: Diastolic blood pressure; eGFR: Estimated glomerular filtration rate; BMI: Body mass index; LDL-C: Low density lipoprotein - cholesterol; GOPC: general outpatient clinic; A&E: Accident & Emergency; SOPC: specialist outpatient clinic.

**Table S3 The Proportion of Patients by UPCI Changes (N=123,665)**

| UPCI change | Number (%) |
| --- | --- |
| 0 | 97,562 (78.89) |
| 0.1%-10% | 5,618 (4.54) |
| 10.1%-20% | 13,588 (10.99) |
| 20.1%-30% | 2,880 (2.33) |
| 30.1%-40% | 2,399 (1.94) |
| 40.1%-50% | 1,299 (1.05) |
| 50.1%-60% | 208 (0.17) |
| 60.1%-70% | 101 (0.08) |
| 70.1%-80% | 9 (0.01) |
| ≥80.1% | 1 (0.00) |

UPCI: Usual provider continuity index.

**Table S4 Data Completion Rates of the Baseline Characteristics among Patients with DM (N=123,665)**

| **Clinical parameters** | **N (%)** |
| --- | --- |
|  |  |
| HbA1c (%) | 121,214 (98.0%) |
| SBP (mmHg) | 121,885 (98.6%) |
| DBP (mmHg) | 121,885 (98.6%) |
| eGFR (mL/min/1.73 m^2^) | 121,775 (98.5%) |
| BMI (kg/m^2^) | 108,929 (88.1%) |
| LDL-C (mmol/L) | 121,294 (98.1%) |
| Overall | 107,125 (86.6%) |

DM: diabetes mellitus; HbA1c: glycated hemoglobin; SBP: systolic blood pressure; DBP: diastolic blood pressure; eGFR: estimated glomerular filtration rate; BMI: body mass index; LDL-C: low-density lipoprotein cholesterol.

**Table S5 Associations between Clinic-Based UPCI Reduction and CVD, Kidney Failure and All-Cause Mortality among Patients with DM in Sensitivity Analysis of Using Inverse Probability of Treatment Weighting (N = 124,002)**

| **Outcomes** | **Event** | **Person-year** | **Incidence rate (cases, 1000 person-year)** | **Adjusted HR (95% CI)** |
| --- | --- | --- | --- | --- |
| **CVD** |  |  |  |  |
| UPCI reduction (≤ 10.0%) | 4,481 | 209,546 | 21.38 (20.76,22.01) | Reference |
| UPCI reduction (10.1%-20.0%) | 621 | 27,341 | 22.71 (20.91,24.52) | 1.05 (0.96,1.14) |
| UPCI reduction (20.1%-30.0%) | 151 | 5,378 | 28.08 (23.50,32.66) | 1.27 (1.08,1.50)** |
| UPCI reduction (≥30.1%) | 218 | 7,657 | 28.47 (24.62,32.32) | 1.28 (1.11,1.47)*** |
| **CHD** |  |  |  |  |
| UPCI reduction (≤ 10.0%) | 2,170 | 211,482 | 10.26 (9.83,10.70) | Reference |
| UPCI reduction (10.1%-20.0%) | 303 | 27,600 | 10.98 (9.72,12.23) | 1.04 (0.92,1.18) |
| UPCI reduction (20.1%-30.0%) | 78 | 5,440 | 14.34 (11.06,17.62) | 1.32 (1.05,1.66)* |
| UPCI reduction (≥30.1%) | 108 | 7,753 | 13.93 (11.23,16.62) | 1.26 (1.03,1.54)* |
| **HF** |  |  |  |  |
| UPCI reduction (≤ 10.0%) | 843 | 212,813 | 3.96 (3.69,4.23) | Reference |
| UPCI reduction (10.1%-20.0%) | 117 | 27,769 | 4.21 (3.43,5.00) | 1.11 (0.92,1.35) |
| UPCI reduction (20.1%-30.0%) | 25 | 5,480 | 4.56 (2.67,6.45) | 1.34 (0.92,1.94) |
| UPCI reduction (≥30.1%) | 44 | 7,795 | 5.64 (3.91,7.38) | 1.68 (1.27,2.23)*** |
| **Stroke** |  |  |  |  |
| UPCI reduction (≤ 10.0%) | 1,767 | 211,968 | 8.34 (7.95,8.73) | Reference |
| UPCI reduction (10.1%-20.0%) | 248 | 27,661 | 8.97 (7.83,10.10) | 1.05 (0.92,1.20) |
| UPCI reduction (20.1%-30.0%) | 55 | 5,450 | 10.09 (7.32,12.86) | 1.17 (0.89,1.53) |
| UPCI reduction (≥30.1%) | 79 | 7,774 | 10.16 (7.85,12.47) | 1.10 (0.87,1.40) |
| **Kidney failure** |  |  |  |  |
| UPCI reduction (≤ 10.0%) | 1,399 | 212,655 | 6.58 (6.23,6.93) | Reference |
| UPCI reduction (10.1%-20.0%) | 154 | 27,764 | 5.55 (4.65,6.44) | 0.91 (0.77,1.06) |
| UPCI reduction (20.1%-30.0%) | 52 | 5,468 | 9.51 (6.83,12.19) | 1.47 (1.12,1.94)** |
| UPCI reduction (≥30.1%) | 76 | 7,800 | 9.74 (7.48,12.00) | 1.32 (1.03,1.68)* |
| **All-cause mortality** |  |  |  |  |
| UPCI reduction (≤ 10.0%) | 3,026 | 213,445 | 14.18 (13.67,14.68) | Reference |
| UPCI reduction (10.1%-20.0%) | 410 | 27,858 | 14.72 (13.27,16.16) | 1.01 (0.91,1.12) |
| UPCI reduction (20.1%-30.0%) | 93 | 5,499 | 16.91 (13.38,20.45) | 1.11 (0.90,1.37) |
| UPCI reduction (≥30.1%) | 141 | 7,836 | 17.99 (14.96,21.03) | 1.15 (0.97,1.37) |

*: p < 0.05; **: p < 0.01; ***: p< 0.001

Hazard ratios were adjusted for age, sex, Charlson Comorbidity Index, smoking, systolic blood pressure, diastolic blood pressure, glycated hemoglobin, estimated glomerular filtration rate, body mass index, low-density lipoprotein cholesterol, insulin, oral antidiabetic drugs, lipid lowering drugs, antihypertensive drugs, UPCI (August 2017 - December 2019), number of hospital admissions (January 2020- May 2022), number of A&E attendance (January 2020- May 2022) and number of SOPC attendance (January 2020- May 2022)..

GOPC: general outpatient clinic; DM: diabetes mellitus; UPCI: Usual provider continuity index; CVD: cardiovascular disease; CHD: coronary heart disease; HF: heart failure.

**Table S6 Associations of Clinic-Based UPCI Reduction with CVD and Kidney Failure among Patients with DM in Sensitivity Analysis using Competing Risk Model (N = 123,403)**

| **Outcomes** | **Event** | **Person-year** | **Incidence rate (cases, 1000 person-year)** | **Adjusted HR (95% CI)** |
| --- | --- | --- | --- | --- |
| **CVD** |  |  |  |  |
| UPCI reduction (≤ 10.0%) | 4,484 | 207,568 | 21.60 (20.97,22.25) | Reference |
| UPCI reduction (10.1%-20.0%) | 620 | 27,307 | 22.69 (20.94,24.62) | 1.05 (0.97,1.14) |
| UPCI reduction (20.1%-30.0%) | 165 | 5,749 | 28.76 (23.62,35.37) | 1.33 (1.14,1.56)*** |
| UPCI reduction (≥30.1%) | 225 | 8,043 | 28.02 (23.87,33.12) | 1.30 (1.13,1.48)*** |
| **CHD** |  |  |  |  |
| UPCI reduction (≤ 10.0%) | 2,176 | 209,491 | 10.39 (9.96,10.84) | Reference |
| UPCI reduction (10.1%-20.0%) | 299 | 27,566 | 10.85 (9.67,12.21) | 1.04 (0.93,1.18) |
| UPCI reduction (20.1%-30.0%) | 79 | 5,820 | 13.65 (10.42,18.23) | 1.31 (1.05,1.64)* |
| UPCI reduction (≥30.1%) | 111 | 8,143 | 13.61 (10.75,17.49) | 1.31 (1.08,1.58)* |
| **HF** |  |  |  |  |
| UPCI reduction (≤ 10.0%) | 832 | 210,819 | 3.94 (3.69,4.23) | Reference |
| UPCI reduction (10.1%-20.0%) | 121 | 27,736 | 4.36 (3.64,5.29) | 1.11 (0.91,1.34) |
| UPCI reduction (20.1%-30.0%) | 40 | 5,858 | 6.78 (4.22,11.61) | 1.72 (1.25,2.36)** |
| UPCI reduction (≥30.1%) | 56 | 8,190 | 6.89 (4.92,9.95) | 1.74 (1.33,2.28)*** |
| **Stroke** |  |  |  |  |
| UPCI reduction (≤ 10.0%) | 1,772 | 209,990 | 8.44 (8.05,8.85) | Reference |
| UPCI reduction (10.1%-20.0%) | 246 | 27,629 | 8.90 (7.84,10.14) | 1.06 (0.92,1.21) |
| UPCI reduction (20.1%-30.0%) | 57 | 5,831 | 9.82 (7.23,13.69) | 1.16 (0.89,1.51) |
| UPCI reduction (≥30.1%) | 75 | 8,172 | 9.20 (7.06,12.22) | 1.09 (0.86,1.37) |
| **Kidney failure** |  |  |  |  |
| UPCI reduction (≤ 10.0%) | 1,375 | 210,671 | 6.53 (6.19,6.89) | Reference |
| UPCI reduction (10.1%-20.0%) | 163 | 27,732 | 5.87 (5.00,6.92) | 0.90 (0.76,1.06) |
| UPCI reduction (20.1%-30.0%) | 62 | 5,840 | 10.70 (7.69,15.33) | 1.63 (1.27,2.11)*** |
| UPCI reduction (≥30.1%) | 70 | 8,199 | 8.54 (6.48,11.50) | 1.31 (1.03,1.66)* |

*: p < 0.05; **: p < 0.01; ***: p< 0.001.

Hazard ratios were adjusted for age, sex, Charlson Comorbidity Index, smoking, systolic blood pressure, diastolic blood pressure, glycated hemoglobin, estimated glomerular filtration rate, body mass index, low-density lipoprotein cholesterol, insulin, oral antidiabetic drugs, lipid lowering drugs, antihypertensive drugs, UPCI (August 2017 - December 2019), number of hospital admissions (January 2020- May 2022), number of A&E attendance (January 2020- May 2022) and number of SOPC attendance (January 2020- May 2022).

GOPC: general outpatient clinic; DM: diabetes mellitus; UPCI: Usual provider continuity index; CVD: cardiovascular disease; CHD: coronary heart disease; HF: heart failure.

**Table S7 Associations of Clinic-Based UPCI Reduction with CVD, Kidney Failure and All-Cause Mortality among Patients with DM in Sensitivity Analysis of including patients with at least five GOPC Attendance (N = 115,844)**

| **Outcomes** | **Event** | **Person-year** | **Incidence rate (cases, 1000 person-year)** | **Adjusted HR (95% CI)** |
| --- | --- | --- | --- | --- |
| **CVD** |  |  |  |  |
| UPCI reduction (≤ 10.0%) | 4,195 | 195,518 | 21.46 (20.81,22.13) | Reference |
| UPCI reduction (10.1%-20.0%) | 592 | 26,436 | 22.39 (20.64,24.33) | 1.04 (0.96,1.14) |
| UPCI reduction (20.1%-30.0%) | 134 | 4,852 | 27.62 (22.46,34.34) | 1.29 (1.08,1.53)** |
| UPCI reduction (≥30.1%) | 184 | 6,723 | 27.30 (23.06,32.56) | 1.27 (1.10,1.48)** |
| **CHD** |  |  |  |  |
| UPCI reduction (≤ 10.0%) | 2,055 | 197,317 | 10.41 (9.97,10.88) | Reference |
| UPCI reduction (10.1%-20.0%) | 288 | 26,683 | 10.80 (9.61,12.17) | 1.04 (0.92,1.17) |
| UPCI reduction (20.1%-30.0%) | 63 | 4,911 | 12.77 (9.53,17.52) | 1.23 (0.95,1.58) |
| UPCI reduction (≥30.1%) | 85 | 6,809 | 12.49 (9.77,16.24) | 1.20 (0.97,1.49) |
| **HF** |  |  |  |  |
| UPCI reduction (≤ 10.0%) | 760 | 198,595 | 3.83 (3.57,4.11) | Reference |
| UPCI reduction (10.1%-20.0%) | 116 | 26,845 | 4.32 (3.59,5.26) | 1.13 (0.93,1.37) |
| UPCI reduction (20.1%-30.0%) | 31 | 4,938 | 6.29 (3.90,10.83) | 1.64 (1.15,2.35)** |
| UPCI reduction (≥30.1%) | 46 | 6,852 | 6.67 (4.67,9.88) | 1.74 (1.29,2.35)*** |
| **Stroke** |  |  |  |  |
| UPCI reduction (≤ 10.0%) | 1,648 | 197,803 | 8.33 (7.94,8.75) | Reference |
| UPCI reduction (10.1%-20.0%) | 233 | 26,746 | 8.71 (7.65,9.95) | 1.05 (0.91,1.20) |
| UPCI reduction (20.1%-30.0%) | 48 | 4,915 | 9.83 (7.11,14.01) | 1.18 (0.89,1.57) |
| UPCI reduction (≥30.1%) | 64 | 6,833 | 9.44 (7.17,12.68) | 1.13 (0.88,1.45) |
| **Kidney failure** |  |  |  |  |
| UPCI reduction (≤ 10.0%) | 1,240 | 198,480 | 6.25 (5.91,6.61) | Reference |
| UPCI reduction (10.1%-20.0%) | 154 | 26,845 | 5.75 (4.89,6.82) | 0.92 (0.78,1.09) |
| UPCI reduction (20.1%-30.0%) | 52 | 4,926 | 10.58 (7.60,15.17) | 1.69 (1.28,2.24)*** |
| UPCI reduction (≥30.1%) | 47 | 6,864 | 6.82 (4.94,9.70) | 1.09 (0.82,1.46) |
| **All-cause mortality** |  |  |  |  |
| UPCI reduction (≤ 10.0%) | 2,807 | 199,174 | 14.09 (13.58,14.64) | Reference |
| UPCI reduction (10.1%-20.0%) | 386 | 26,933 | 14.32 (12.95,15.87) | 1.02 (0.91,1.13) |
| UPCI reduction (20.1%-30.0%) | 81 | 4,958 | 16.31 (12.54,21.60) | 1.16 (0.93,1.44) |
| UPCI reduction (≥30.1%) | 110 | 6,888 | 15.93 (12.82,20.06) | 1.13 (0.93,1.37) |

*: p < 0.05; **: p < 0.01; ***: p< 0.001

Hazard ratios were adjusted for age, sex, Charlson Comorbidity Index, smoking, systolic blood pressure, diastolic blood pressure, glycated hemoglobin, estimated glomerular filtration rate, body mass index, low-density lipoprotein cholesterol, insulin, oral antidiabetic drugs, lipid lowering drugs, antihypertensive drugs, UPCI (August 2017 - December 2019), number of hospital admissions (January 2020- May 2022), number of A&E attendance (January 2020- May 2022) and number of SOPC attendance (January 2020- May 2022).

GOPC: general outpatient clinic; DM: diabetes mellitus; UPCI: Usual provider continuity index; CVD: cardiovascular disease; CHD: coronary heart disease; HF: heart failure

**Table S8** **Associations of Clinic-Based UPCI Reduction with CVD, Kidney Failure and All-Cause Mortality among Patients with DM in Sensitivity Analysis of using UPCI Changes (per 10% decrease) as a Continuous Variable (N = 120,008)**

| **Outcomes** | **Adjusted HR (95% CI)** |
| --- | --- |
| **CVD** | 1.08 (1.05,1.11)*** |
| CHD | 1.08 (1.03,1.13)** |
| HF | 1.15 (1.07,1.24)*** |
| Stroke | 1.04 (0.99,1.09) |
| **Kidney failure** | 1.08 (1.02,1.14)* |
| **All-cause mortality** | 1.05 (1.01,1.09)* |

*: p < 0.05; **: p < 0.01; ***: p< 0.001

Hazard ratios were adjusted for age, sex, Charlson Comorbidity Index, smoking, systolic blood pressure, diastolic blood pressure, glycated hemoglobin, estimated glomerular filtration rate, body mass index, low-density lipoprotein cholesterol, insulin, oral antidiabetic drugs, lipid lowering drugs, antihypertensive drugs, UPCI (August 2017 - December 2019), number of hospital admissions (January 2020- May 2022), number of A&E attendance (January 2020- May 2022) and number of SOPC attendance (January 2020- May 2022).

DM: diabetes mellitus; UPCI: Usual provider continuity index; CVD: cardiovascular disease; CHD: coronary heart disease; HF: heart failure.

**Table S9 Associations of Clinic-Based UPCI Reduction with Kidney Failure among Patients with DM in Sensitivity Analysis of Using at Least Two eGFR Measurements <15 mL/min/1.73 m² (N = 123,403)**

| **Outcomes** | **Event** | **Person-year** | **Incidence rate (cases, 1000 person-year)** | **Adjusted HR (95% CI)** |
| --- | --- | --- | --- | --- |
| **Kidney failure** |  |  |  |  |
| UPCI change (≤ 10%) | 1,117 | 210,820 | 5.30 (4.99,5.62) | Reference |
| UPCI change (10.1%-20%) | 134 | 27,747 | 4.84 (4.06,5.81) | 0.91 (0.76,1.09) |
| UPCI change (20.1%-30%) | 50 | 5,847 | 8.62 (5.94,13.03) | 1.63 (1.23,2.16)*** |
| UPCI change (≥30.1%) | 60 | 8,211 | 7.33 (5.43,10.16) | 1.38 (1.07,1.79)* |

*: p < 0.05; ***: p< 0.001

Hazard ratios were adjusted for age, sex, Charlson Comorbidity Index, smoking, systolic blood pressure, diastolic blood pressure, glycated hemoglobin, estimated glomerular filtration rate, body mass index, low-density lipoprotein cholesterol, insulin, oral antidiabetic drugs, lipid lowering drugs, antihypertensive drugs, UPCI (August 2017 - December 2019), number of hospital admissions (January 2020- May 2022), number of A&E attendance (January 2020- May 2022) and number of SOPC attendance (January 2020- May 2022).

DM: diabetes mellitus; UPCI: Usual provider continuity index, eGFR: estimated glomerular filtration rate.

**Table S10 Associations of Clinic-Based UPCI Reduction with CVD, Kidney Failure and All-Cause Mortality among Patients with DM in Sensitivity Analysis of adjusting HbA1c, Blood Pressure, and Medication Use as Time-varying Confounders (N = 123,403)**

| **Outcomes** | **Event** | **Person-year** | **Incidence rate (cases, 1000 person-year)** | **Adjusted HR (95% CI)** |
| --- | --- | --- | --- | --- |
| **CVD** |  |  |  |  |
| UPCI change (≤ 10%) | 4,484 | 207,500 | 21.60 (20.40,22.80) | Reference |
| UPCI change (10.1%-20%) | 620 | 27,307 | 22.80 (20.40,25.20) | 1.04 (0.95,1.13) |
| UPCI change (20.1%-30%) | 165 | 5,749 | 28.80 (24.00,34.80) | 1.31 (1.12,1.53)** |
| UPCI change (≥30.1%) | 225 | 8,043 | 27.60 (24.00,33.60) | 1.27 (1.11,1.45)** |
| **CHD** |  |  |  |  |
| UPCI change (≤ 10%) | 2,176 | 209,167 | 10.80 (9.60,10.80) | Reference |
| UPCI change (10.1%-20%) | 299 | 27,566 | 10.80 (9.60,12.00) | 1.03 (0.91,1.16) |
| UPCI change (20.1%-30%) | 79 | 5,820 | 13.20 (10.80,18.00) | 1.34 (1.07,1.68)* |
| UPCI change (≥30.1%) | 111 | 8,143 | 13.20 (10.80,18.00) | 1.33 (1.10,1.61)** |
| **HF** |  |  |  |  |
| UPCI change (≤ 10%) | 832 | 210,833 | 3.60 (3.60,4.80) | Reference |
| UPCI change (10.1%-20%) | 121 | 27,736 | 4.80 (3.60,4.80) | 1.18 (0.98,1.43) |
| UPCI change (20.1%-30%) | 40 | 5,858 | 7.20 (4.80,12.00) | 1.45 (1.05,1.99)* |
| UPCI change (≥30.1%) | 56 | 8,190 | 7.20 (4.80,9.60) | 1.56 (1.19,2.05)** |
| **Stroke** |  |  |  |  |
| UPCI change (≤ 10%) | 1,772 | 210,000 | 8.40 (8.40,8.40) | Reference |
| UPCI change (10.1%-20%) | 246 | 27,629 | 8.40 (8.40,9.60) | 1.03 (0.90,1.18) |
| UPCI change (20.1%-30%) | 57 | 5,831 | 9.60 (7.20,13.20) | 1.13 (0.86,1.46) |
| UPCI change (≥30.1%) | 75 | 8,172 | 9.60 (7.20,12.00) | 1.04 (0.82,1.31) |
| **Kidney failure** |  |  |  |  |
| UPCI change (≤ 10%) | 1,375 | 210,833 | 6.00 (6.00,7.20) | Reference |
| UPCI change (10.1%-20%) | 163 | 27,732 | 6.00 (4.80,7.20) | 0.93 (0.79,1.09) |
| UPCI change (20.1%-30%) | 62 | 5,840 | 10.80 (7.20,15.60) | 1.12 (0.87,1.45) |
| UPCI change (≥30.1%) | 70 | 8,199 | 8.40 (6.00,12.00) | 1.02 (0.80,1.30) |
| **All-cause mortality** |  |  |  |  |
| UPCI change (≤ 10%) | 3,043 | 211,667 | 14.40 (14.40,14.40) | Reference |
| UPCI change (10.1%-20%) | 402 | 27,826 | 14.40 (13.20,15.60) | 0.99 (0.89,1.10) |
| UPCI change (20.1%-30%) | 97 | 5,882 | 16.80 (13.20,21.60) | 0.84 (0.69,1.03) |
| UPCI change (≥30.1%) | 135 | 8,236 | 16.80 (13.20,20.40) | 0.96 (0.81,1.14) |

*: p < 0.05; **: p < 0.01; ***: p< 0.001

Hazard ratios were adjusted for age, sex, Charlson Comorbidity Index, smoking, systolic blood pressure, diastolic blood pressure, glycated hemoglobin, estimated glomerular filtration rate, body mass index, low-density lipoprotein cholesterol, insulin, oral antidiabetic drugs, lipid lowering drugs, antihypertensive drugs, UPCI (August 2017 - December 2019), number of hospital admissions (January 2020- May 2022), number of A&E attendance (January 2020- May 2022) and number of SOPC attendance (January 2020- May 2022).

DM: diabetes mellitus; UPCI: Usual provider continuity index; CVD: cardiovascular disease; CHD: coronary heart disease; HF: heart failure; HbA1c: glycated hemoglobin.

**Table S11 Associations of Clinic-Based UPCI Reduction with CVD, Kidney Failure and All-Cause Mortality among Patients with DM in Sensitivity Analysis of Including Patients with COVID-19 (N = 160,732)**

| **Outcomes** | **Event** | **Person-year** | **Incidence rate (cases, 1000 person-year)** | **Adjusted HR (95% CI)** |
| --- | --- | --- | --- | --- |
| **CVD** |  |  |  |  |
| UPCI change (≤ 10%) | 6,673 | 264,844 | 25.20 (24.59,25.82) | Reference |
| UPCI change (10.1%-20%) | 992 | 37,011 | 26.81 (25.15,28.61) | 1.06 (1.00,1.14) |
| UPCI change (20.1%-30%) | 256 | 8,413 | 30.47 (26.28,35.53) | 1.21 (1.07,1.37)** |
| UPCI change (≥30.1%) | 367 | 11,862 | 30.94 (27.33,35.17) | 1.23 (1.11,1.36)*** |
| **CHD** |  |  |  |  |
| UPCI change (≤ 10%) | 3,182 | 267,790 | 11.88 (11.47,12.31) | Reference |
| UPCI change (10.1%-20%) | 463 | 37,459 | 12.37 (11.27,13.60) | 1.04 (0.94,1.15) |
| UPCI change (20.1%-30%) | 124 | 8,522 | 14.51 (11.78,18.09) | 1.22 (1.02,1.46)* |
| UPCI change (≥30.1%) | 170 | 12,042 | 14.12 (11.80,17.06) | 1.19 (1.02,1.39)* |
| **HF** |  |  |  |  |
| UPCI change (≤ 10%) | 1,340 | 269,632 | 4.97 (4.71,5.25) | Reference |
| UPCI change (10.1%-20%) | 203 | 37,708 | 5.38 (4.67,6.23) | 1.08 (0.93,1.25) |
| UPCI change (20.1%-30%) | 60 | 8,580 | 6.95 (5.05,9.84) | 1.40 (1.08,1.81)* |
| UPCI change (≥30.1%) | 75 | 12,139 | 6.22 (4.68,8.44) | 1.25 (0.99,1.58) |
| **Stroke** |  |  |  |  |
| UPCI change (≤ 10%) | 2,625 | 268,424 | 9.78 (9.41,10.17) | Reference |
| UPCI change (10.1%-20%) | 401 | 37,533 | 10.69 (9.67,11.85) | 1.09 (0.98,1.21) |
| UPCI change (20.1%-30%) | 96 | 8,535 | 11.22 (8.83,14.48) | 1.15 (0.94,1.41) |
| UPCI change (≥30.1%) | 150 | 12,062 | 12.41 (10.22,15.22) | 1.27 (1.08,1.50)*** |
| **Kidney failure** |  |  |  |  |
| UPCI change (≤ 10%) | 2,214 | 269,349 | 8.22 (7.88,8.58) | Reference |
| UPCI change (10.1%-20%) | 288 | 37,698 | 7.64 (6.78,8.65) | 0.93 (0.82,1.05) |
| UPCI change (20.1%-30%) | 90 | 8,562 | 10.54 (8.28,13.64) | 1.28 (1.04,1.58)* |
| UPCI change (≥30.1%) | 125 | 12,122 | 10.30 (8.32,12.90) | 1.25 (1.05,1.50)* |
| **All-cause mortality** |  |  |  |  |
| UPCI change (≤ 10%) | 4,741 | 270,642 | 17.52 (17.02,18.04) | Reference |
| UPCI change (10.1%-20%) | 658 | 37,866 | 17.37 (16.07,18.80) | 0.99 (0.91,1.08) |
| UPCI change (20.1%-30%) | 149 | 8,619 | 17.25 (14.22,21.13) | 0.98 (0.84,1.16) |
| UPCI change (≥30.1%) | 246 | 12,198 | 20.19 (17.33,23.66) | 1.15 (1.01,1.31)* |

*: p < 0.05; **: p < 0.01; ***: p< 0.001

Hazard ratios were adjusted for age, sex, Charlson Comorbidity Index, COVID-19, smoking, systolic blood pressure, diastolic blood pressure, glycated hemoglobin, estimated glomerular filtration rate, body mass index, low-density lipoprotein cholesterol, insulin, oral antidiabetic drugs, lipid lowering drugs, antihypertensive drugs, UPCI (August 2017 - December 2019), number of hospital admissions (January 2020- May 2022), number of A&E attendance (January 2020- May 2022) and number of SOPC attendance (January 2020- May 2022).

DM: diabetes mellitus; UPCI: Usual provider continuity index; CVD: cardiovascular disease; CHD: coronary heart disease; HF: heart failure.

**Table S12 Associations of Clinic-Based UPCI Reduction with CVD, Kidney Failure and All-Cause Mortality among Patients with DM in Sensitivity Analysis of Using Pre-outbreak Variables to Construct Propensity Scores (N = 123,429)**

| **Outcomes** | **Event** | **Person-year** | **Incidence rate (cases, 1000 person-year)** | **Adjusted HR (95% CI)** |
| --- | --- | --- | --- | --- |
| **CVD** |  |  |  |  |
| UPCI change (≤ 10%) | 4,461 | 207,694 | 21.48 (20.85,22.13) | Reference |
| UPCI change (10.1%-20%) | 623 | 27,315 | 22.82 (21.07,24.75) | 1.06 (0.98,1.16) |
| UPCI change (20.1%-30%) | 164 | 5,751 | 28.57 (23.82,34.57) | 1.33 (1.14,1.56)*** |
| UPCI change (≥30.1%) | 229 | 8,021 | 28.58 (24.57,33.45) | 1.33 (1.17,1.52)*** |
| **CHD** |  |  |  |  |
| UPCI change (≤ 10%) | 2,170 | 209,607 | 10.35 (9.92,10.80) | Reference |
| UPCI change (10.1%-20%) | 299 | 27,577 | 10.85 (9.67,12.20) | 1.05 (0.93,1.18) |
| UPCI change (20.1%-30%) | 79 | 5,821 | 13.56 (10.50,17.81) | 1.31 (1.05,1.64)* |
| UPCI change (≥30.1%) | 110 | 8,126 | 13.53 (10.88,17.02) | 1.31 (1.08,1.58)** |
| **HF** |  |  |  |  |
| UPCI change (≤ 10%) | 825 | 210,933 | 3.91 (3.65,4.19) | Reference |
| UPCI change (10.1%-20%) | 122 | 27,750 | 4.41 (3.67,5.33) | 1.13 (0.93,1.36) |
| UPCI change (20.1%-30%) | 36 | 5,859 | 6.15 (4.07,9.76) | 1.57 (1.13,2.20)** |
| UPCI change (≥30.1%) | 51 | 8,172 | 6.29 (4.55,8.94) | 1.61 (1.21,2.13)*** |
| **Stroke** |  |  |  |  |
| UPCI change (≤ 10%) | 1,761 | 210,107 | 8.38 (8.00,8.79) | Reference |
| UPCI change (10.1%-20%) | 249 | 27,641 | 9.01 (7.95,10.26) | 1.07 (0.94,1.23) |
| UPCI change (20.1%-30%) | 60 | 5,829 | 10.23 (7.60,14.11) | 1.22 (0.94,1.58) |
| UPCI change (≥30.1%) | 82 | 8,144 | 10.03 (7.80,13.12) | 1.20 (0.96,1.49) |
| **Kidney failure** |  |  |  |  |
| UPCI change (≤ 10%) | 1,370 | 210,783 | 6.50 (6.17,6.86) | Reference |
| UPCI change (10.1%-20%) | 164 | 27,745 | 5.92 (5.05,6.99) | 0.91 (0.78,1.07) |
| UPCI change (20.1%-30%) | 60 | 5,846 | 10.29 (7.61,14.27) | 1.58 (1.22,2.05)*** |
| UPCI change (≥30.1%) | 75 | 8,173 | 9.12 (7.01,12.10) | 1.40 (1.11,1.77)** |
| **All-cause mortality** |  |  |  |  |
| UPCI change (≤ 10%) | 2,989 | 211,563 | 14.13 (13.63,14.65) | Reference |
| UPCI change (10.1%-20%) | 410 | 27,840 | 14.72 (13.34,16.28) | 1.04 (0.94,1.16) |
| UPCI change (20.1%-30%) | 105 | 5,881 | 17.89 (14.22,22.82) | 1.27 (1.04,1.54)* |
| UPCI change (≥30.1%) | 149 | 8,214 | 18.12 (15.01,22.08) | 1.28 (1.09,1.51)** |

*: p < 0.05; **: p < 0.01; ***: p< 0.001

Hazard ratios were adjusted for age, sex, Charlson Comorbidity Index, smoking, systolic blood pressure, diastolic blood pressure, glycated hemoglobin, estimated glomerular filtration rate, body mass index, low-density lipoprotein cholesterol, insulin, oral antidiabetic drugs, lipid lowering drugs, antihypertensive drugs, UPCI (August 2017 - December 2019), number of hospital admissions (August 2017 - December 2019), number of A&E attendance (August 2017 - December 2019) and number of SOPC attendance (August 2017 - December 2019).

DM: diabetes mellitus; UPCI: Usual provider continuity index; CVD: cardiovascular disease; CHD: coronary heart disease; HF: heart failure.

**Table S13 Associations of Clinic-Based UPCI Reduction with CVD, Kidney Failure and All-Cause Mortality among Patients with DM in Sensitivity Analysis of Removing Healthcare Utilization during the Outbreak Period in the Adjustment Sets (N = 123,416)**

| **Outcomes** | **Event** | **Person-year** | **Incidence rate (cases, 1000 person-year)** | **Adjusted HR (95% CI)** |
| --- | --- | --- | --- | --- |
| **CVD** |  |  |  |  |
| UPCI change (≤ 10%) | 4,458 | 210,182 | 21.21 (20.59,21.85) | Reference |
| UPCI change (10.1%-20%) | 626 | 27,614 | 22.66 (20.92,24.59) | 1.07 (0.98,1.16) |
| UPCI change (20.1%-30%) | 160 | 5,814 | 27.44 (22.77,33.36) | 1.29 (1.11,1.52)** |
| UPCI change (≥30.1%) | 223 | 8,157 | 27.28 (23.44,31.93) | 1.29 (1.12,1.47)*** |
| **CHD** |  |  |  |  |
| UPCI change (≤ 10%) | 2,171 | 212,382 | 10.22 (9.80,10.67) | Reference |
| UPCI change (10.1%-20%) | 300 | 27,927 | 10.75 (9.59,12.10) | 1.05 (0.93,1.19) |
| UPCI change (20.1%-30%) | 79 | 5,908 | 13.36 (10.30,17.63) | 1.31 (1.04,1.64)* |
| UPCI change (≥30.1%) | 106 | 8,265 | 12.84 (10.32,16.17) | 1.26 (1.03,1.53)* |
| **HF** |  |  |  |  |
| UPCI change (≤ 10%) | 822 | 213,774 | 3.84 (3.59,4.12) | Reference |
| UPCI change (10.1%-20%) | 121 | 28,115 | 4.29 (3.57,5.20) | 1.12 (0.92,1.35) |
| UPCI change (20.1%-30%) | 30 | 5,948 | 5.09 (3.26,8.40) | 1.32 (0.92,1.90) |
| UPCI change (≥30.1%) | 52 | 8,313 | 6.28 (4.53,8.95) | 1.63 (1.23,2.16)*** |
| **Stroke** |  |  |  |  |
| UPCI change (≤ 10%) | 1,761 | 212,863 | 8.27 (7.89,8.68) | Reference |
| UPCI change (10.1%-20%) | 251 | 27,981 | 8.97 (7.91,10.21) | 1.08 (0.95,1.24) |
| UPCI change (20.1%-30%) | 60 | 5,914 | 10.18 (7.54,14.08) | 1.23 (0.95,1.59) |
| UPCI change (≥30.1%) | 76 | 8,289 | 9.18 (7.15,11.99) | 1.11 (0.88,1.40) |
| **Kidney failure** |  |  |  |  |
| UPCI change (≤ 10%) | 1,360 | 213,224 | 6.38 (6.05,6.73) | Reference |
| UPCI change (10.1%-20%) | 165 | 28,075 | 5.89 (5.02,6.95) | 0.92 (0.79,1.09) |
| UPCI change (20.1%-30%) | 56 | 5,933 | 9.40 (6.94,13.05) | 1.47 (1.13,1.93)** |
| UPCI change (≥30.1%) | 72 | 8,291 | 8.72 (6.66,11.65) | 1.37 (1.08,1.73)** |
| **All-cause mortality** |  |  |  |  |
| UPCI change (≤ 10%) | 2,982 | 211,567 | 14.10 (13.60,14.62) | Reference |
| UPCI change (10.1%-20%) | 409 | 27,817 | 14.72 (13.34,16.28) | 1.04 (0.94,1.16) |
| UPCI change (20.1%-30%) | 104 | 5,875 | 17.75 (14.08,22.72) | 1.26 (1.04,1.53)* |
| UPCI change (≥30.1%) | 138 | 8,226 | 16.77 (13.84,20.51) | 1.19 (1.00,1.41)* |

*: p < 0.05; **: p < 0.01; ***: p< 0.001.

Hazard ratios were adjusted for age, sex, Charlson Comorbidity Index, smoking, systolic blood pressure, diastolic blood pressure, glycated hemoglobin, estimated glomerular filtration rate, body mass index, low-density lipoprotein cholesterol, insulin, oral antidiabetic drugs, lipid lowering drugs, antihypertensive drugs, UPCI (August 2017 - December 2019).

DM: diabetes mellitus; UPCI: Usual provider continuity index; CVD: cardiovascular disease; CHD: coronary heart disease; HF: heart failure.

**Table S14 Associations of Clinic-Based UPCI Reduction with CVD, Kidney Failure and All-Cause Mortality among Patients with DM in Sensitivity Analysis of Excluding Individuals Diagnosed with Pneumonia, Cough, and Upper Respiratory Infection (N = 121,243)**

| **Outcomes** | **Event** | **Person-year** | **Incidence rate (cases, 1000 person-year)** | **Adjusted HR (95% CI)** |
| --- | --- | --- | --- | --- |
| **CVD** |  |  |  |  |
| UPCI change (≤ 10%) | 4,379 | 204,416 | 21.42 (20.79,22.08) | Reference |
| UPCI change (10.1%-20%) | 599 | 26,684 | 22.43 (20.68,24.38) | 1.05 (0.96,1.14) |
| UPCI change (20.1%-30%) | 157 | 5,519 | 28.52 (23.32,35.25) | 1.33 (1.14,1.56)*** |
| UPCI change (≥30.1%) | 212 | 7,761 | 27.37 (23.28,32.41) | 1.28 (1.11,1.47)*** |
| **CHD** |  |  |  |  |
| UPCI change (≤ 10%) | 2,137 | 206,284 | 10.36 (9.92,10.82) | Reference |
| UPCI change (10.1%-20%) | 285 | 26,939 | 10.57 (9.40,11.93) | 1.02 (0.90,1.15) |
| UPCI change (20.1%-30%) | 74 | 5,586 | 13.33 (10.04,18.08) | 1.29 (1.02,1.62)* |
| UPCI change (≥30.1%) | 104 | 7,855 | 13.23 (10.49,16.93) | 1.28 (1.05,1.55)* |
| **HF** |  |  |  |  |
| UPCI change (≤ 10%) | 789 | 207,610 | 3.80 (3.55,4.08) | Reference |
| UPCI change (10.1%-20%) | 116 | 27,097 | 4.29 (3.57,5.22) | 1.13 (0.93,1.37) |
| UPCI change (20.1%-30%) | 36 | 5,623 | 6.38 (3.98,10.89) | 1.68 (1.20,2.35)** |
| UPCI change (≥30.1%) | 53 | 7,899 | 6.72 (4.76,9.80) | 1.77 (1.34,2.33)*** |
| **Stroke** |  |  |  |  |
| UPCI change (≤ 10%) | 1,738 | 206,779 | 8.41 (8.02,8.82) | Reference |
| UPCI change (10.1%-20%) | 243 | 26,988 | 9.00 (7.92,10.26) | 1.07 (0.94,1.22) |
| UPCI change (20.1%-30%) | 55 | 5,599 | 9.84 (7.15,13.93) | 1.17 (0.90,1.53) |
| UPCI change (≥30.1%) | 71 | 7,881 | 8.98 (6.83,12.07) | 1.07 (0.84,1.36) |
| **Kidney failure** |  |  |  |  |
| UPCI change (≤ 10%) | 1,350 | 207,450 | 6.51 (6.17,6.87) | Reference |
| UPCI change (10.1%-20%) | 159 | 27,092 | 5.88 (5.00,6.95) | 0.90 (0.77,1.06) |
| UPCI change (20.1%-30%) | 56 | 5,610 | 9.97 (7.17,14.30) | 1.53 (1.17,2.00)** |
| UPCI change (≥30.1%) | 70 | 7,905 | 8.81 (6.66,11.92) | 1.35 (1.06,1.72)* |
| **All-cause mortality** |  |  |  |  |
| UPCI change (≤ 10%) | 2,982 | 208,212 | 14.32 (13.81,14.86) | Reference |
| UPCI change (10.1%-20%) | 389 | 27,184 | 14.32 (12.95,15.87) | 1.00 (0.90,1.11) |
| UPCI change (20.1%-30%) | 97 | 5,646 | 17.26 (13.43,22.54) | 1.20 (0.98,1.47) |
| UPCI change (≥30.1%) | 124 | 7,941 | 15.66 (12.76,19.43) | 1.09 (0.91,1.31) |

*: p < 0.05; **: p < 0.01; ***: p< 0.001

Hazard ratios were adjusted for age, sex, Charlson Comorbidity Index, smoking, systolic blood pressure, diastolic blood pressure, glycated hemoglobin, estimated glomerular filtration rate, body mass index, low-density lipoprotein cholesterol, insulin, oral antidiabetic drugs, lipid lowering drugs, antihypertensive drugs, UPCI (August 2017 - December 2019), number of hospital admissions (January 2020- May 2022), number of A&E attendance (January 2020- May 2022) and number of SOPC attendance (January 2020- May 2022).

DM: diabetes mellitus; UPCI: Usual provider continuity index; CVD: cardiovascular disease; CHD: coronary heart disease; HF: heart failure
