# Supplementary material for: Association of Reduction in Continuity of Care During COVID‐19 Pandemic With Cardiovascular Diseases, Kidney Failure and All‐Cause Mortality for People With Diabetes: A Cohort Study in Hong Kong
Source: Diabetes Obes Metab. 2026 Jun 15;28(9):8016–24. doi: 10.1111/dom.70984 (PMC13449042; doi:10.1111/dom.70984)
Supplement: Supplementary file 2 [file DOM-28-8016-s002.docx]

**Figure S1 Directed acyclic graph based on the assumed pathways**

Minimally sufficient adjustment set: Age, BMI, BP, CCI, HbA1c, Healthcare Utilization, LDL-C, Medication use, Pre-outbreak UPCI, Sex, Smoking, eGFR.

Medication use includes insulin, oral antidiabetic drugs, lipid lowering drugs and antihypertensive drugs. BP includes diastolic blood pressure and systolic blood pressure. Healthcare utilization includes number of hospital admissions (January 2020- May 2022), number of A&E attendances (January 2020- May 2022) and number of SOPC attendances (January 2020- May 2022).

BMI: body mass index; CCI: Charlson comorbidity index; BP: blood pressure; eGFR: estimated glomerular filtration rate; HbA1c: glycated hemoglobin; LDL-C: low-density lipoprotein cholesterol; UPCI: usual provider of care index; CVD: cardiovascular diseases.

**Figure S2 Flowchart of patient selection**

DM: diabetes mellitus; CVD: cardiovascular diseases; UPCI: usual provider continuity index; COVID-19: coronavirus disease.

**Figure S3 Subgroup analyses on the association between the UPCI reduction and cardiovascular diseases among patients with DM**

Hazard ratios were adjusted by age, sex, Charlson Comorbidity Index, smoking, systolic blood pressure, diastolic blood pressure, glycated hemoglobin, estimated glomerular filtration rate, body mass index, low-density lipoprotein cholesterol, insulin, oral antidiabetic drugs, lipid lowering drugs, antihypertensive drugs, UPCI (August 2017 - December 2019), number of hospital admissions (January 2020- May 2022), number of A&E attendance (January 2020- May 2022) and number of SOPC attendance (January 2020- May 2022).

BMI: body mass index; CCI: Charlson comorbidity index; DBP: diastolic blood pressure; eGFR: estimated glomerular filtration rate; HbA1c: glycated hemoglobin; HR: hazards ratio; SBP: systolic blood pressure; UPCI: usual provider of care index.
